# Perceptual Sensitivity and Response to Strong Stimuli Are Related

**DOI:** 10.3389/fpsyg.2017.01642

**Published:** 2017-09-22

**Authors:** Anna C. Bolders, Mattie Tops, Guido P. H. Band, Pieter Jan M. Stallen

**Affiliations:** ^1^Cognitive Psychology Unit, Institute of Psychology, Leiden University Leiden, Netherlands; ^2^Leiden Institute for Brain and Cognition, Leiden University Leiden, Netherlands; ^3^Department of Clinical, Neuro- and Developmental Psychology, VU University Amsterdam Amsterdam, Netherlands

**Keywords:** sensitivity, overreactivity, punishment reactivity, reward reactivity, temperament, suppression effect, perception, masked auditory threshold

## Abstract

To shed new light on the long-standing debate about the (in)dependence of sensitivity to weak stimuli and overreactivity to strong stimuli, we examined the relation between these tendencies within the neurobehavioral framework of the Predictive and Reactive Control Systems (PARCS) theory (Tops et al., 2010, 2014). Whereas previous studies only considered overreactivity in terms of the individual tendency to experience unpleasant affect (punishment reactivity) resulting from strong sensory stimulation, we also took the individual tendency to experience pleasant affect (reward reactivity) resulting from strong sensory stimulation into account. According to PARCS theory, these temperamental tendencies overlap in terms of high reactivity toward stimulation, but oppose each other in terms of the response orientation (approach or avoid). PARCS theory predicts that both types of reactivity to strong stimuli relate to sensitivity to weak stimuli, but that these relationships are suppressed due to the opposing relationship between reward and punishment reactivity. We measured punishment and reward reactivity to strong stimuli and sensitivity to weak stimuli using scales from the Adult Temperament Questionnaire (Evans and Rothbart, 2007). Sensitivity was also measured more objectively using the masked auditory threshold. We found that sensitivity to weak stimuli (both self-reported and objectively assessed) was positively associated with self-reported punishment and reward reactivity to strong stimuli, but only when these reactivity measures were controlled for each other, implicating a mutual suppression effect. These results are in line with PARCS theory and suggest that sensitivity to weak stimuli and overreactivity are dependent, but this dependency is likely to be obscured if punishment and reward reactivity are not both taken into account.

## Introduction

It has long been recognized that individuals differ in perceptual sensitivity (Nebylitsyn et al., 1960; Eysenck, 1967). The term sensitivity, however, has multiple meanings. On the one hand sensitivity can be regarded as lower threshold for weak stimuli; on the other hand sensitivity can be conceived as low tolerance or overreactivity to strong stimulation. According to several theorists (Nebylitsyn et al., 1960; Eysenck, 1967; Aron and Aron, 1997) both types of sensitivity arise from the same trait. Thus, individuals with a low perceptual threshold will also have a low level of tolerance for strong stimulation. However, it has also been argued that these two types of sensitivity are independent (Ellermeier et al., 2001; Evans and Rothbart, 2008). To shed new light on this debate we examined the relation between these tendencies within the Predictive and Reactive Control Systems (PARCS) theory (Tops et al., 2010, 2014).

Predictive and Reactive Control Systems theory differentiates two types of reactivity: punishment reactivity and reward reactivity. High punishment reactivity to strong stimuli corresponds to low tolerance for strong stimulation such as noises, light flashes, and odors, and a tendency to experience negative affect from it. High reward reactivity to strong stimuli corresponds to a tendency to derive pleasure from strong stimulation. In PARCS theory these two temperamental tendencies overlap in terms of high reactivity toward stimuli in the environment, but oppose each other in terms of the response orientation (approach or avoid) toward these stimuli. Due to this opposing relationship each type of reactivity can suppress [statistically; see MacKinnon et al. (2000)] the relation between the other type of reactivity and sensitivity to weak stimuli. In the present study we included measures of sensitivity to weak stimuli and of both types of reactivity to be able to test the predicted suppression effects that follow from PARCS theory. This allowed us to investigate whether PARCS theory provides a suitable framework to better understand the dependencies between perceptual sensitivity to weak stimuli and reactivity to strong stimuli.

Before proceeding to the methodological details of our study we will briefly review theories on sensitivity to weak stimuli and reactivity to strong stimuli and evidence supporting these theories. First we will discuss theories that consider sensitivity to weak stimuli and reactivity to strong stimuli as one trait. Then we will discuss theories that regard both traits as independent. Finally we will discuss PARCS theory and our predictions regarding sensitivity and reactivity based on PARCS theory.

### Perceptual Sensitivity and Overreactivity As One Trait

One influential theory that regards sensitivity to weak stimuli and overreactivity to strong stimuli to result from one underlying trait is H. J. Eysenck’s personality theory about extraversion and introversion (Eysenck, 1967). According to Eysenck, introverts have higher arousal levels than extraverts, which causes higher cortical excitability in introverts. Due to their higher cortical excitability, introverts respond more strongly to stimulation than extraverts and have lower thresholds for weak stimulation, but they are also more easily over-aroused by strong stimulation. Because each individual tries to maintain an optimal arousal level, introverts are predicted to seek non-arousing (social) situations, while extraverts seek situations that are highly arousing (Eysenck, 1967). Questionnaires, such as the Eysenck Personality Questionnaire (EPQ; Eysenck and Eysenck, 1975), are based on this prediction and assess the level of Extraversion-Introversion through questions about (social) strategies to maintain optimal arousal level.

A few early empirical studies related Eysenck’s introversion-extraversion personality dimension to sensory sensitivity as measured by threshold performance. Smith (1968) found that higher introversion was indeed associated with lower auditory thresholds for low frequency tones. In addition, Siddle et al. (1969) obtained a significant negative association between extraversion and visual sensitivity as measured by the inverse of the lower absolute threshold. However, neuroticism, another of Eysenck’s personality dimensions, was suggested to be a confounding variable in this study, making it difficult to conclude whether sensitivity arises from extraversion, neuroticism, or from a combination of these two traits. A further limitation of both these studies was that the performance on the psychophysical measures used may have not only depended on actual perceptual sensitivity but also on the criterion for responding. In terms of signal detection theory (Green and Swets, 1966; Macmillan and Creelman, 2005), the criterion for responding reflects how strong the internal signal (e.g., the sensory effect produced by a stimulus) needs to be for an individual to decide that a signal is present. The criterion an individual adopts can differ strongly between situations and tasks and depends, for example, on signal probability or the relative value of correctly detecting or correctly rejecting a signal and the relative cost of missing a signal and falsely reporting a signal. Therefore, Edman et al. (1979) carried out a study using a threshold procedure developed to measured sensitivity independent of the response criterion. They found that introverts had lower detection thresholds for electrocutaneous stimulation, but only when they also scored high on neuroticism. Taken together, these studies provide some support for the idea that sensitivity arises from an underlying trait that may also give rise to over-arousal by strong stimulation. However, the personality questionnaires used in these studies only included questions about (social) strategies to maintain optimal arousal and did not ask about over-arousal by strong stimulation directly. This makes it difficult to draw strong conclusions from these studies concerning the relation between sensitivity to weak stimuli and reactivity to strong stimuli.

Another, more recent, theory that regards sensitivity and overreactivity as belonging to one trait is the highly sensitive person (HSP) theory developed by Aron and Aron (1997). Central to this theory is the trait sensory processing sensitivity (SPS). SPS is regarded as an evolutionary beneficial survival strategy. It is characterized by heightened awareness of subtle external and internal stimuli. While beneficial in certain situations, this trait comes with the cost of getting more easily overwhelmed by stimulating or quickly changing environments (Aron and Aron, 1997; Aron et al., 2012). Aron and Aron (1997) developed the HSP scale as a unidimensional scale to assess SPS. In line with the presumption that sensitivity and reactivity arise from the same trait, the HSP scale includes items that measure sensitivity to subtle stimuli as well as the tendency to get overwhelmed by strong stimulation. However, in a critical analysis of the HSP scale Evans and Rothbart (2008) questioned the unidimensionality of the scale and argued that sensitivity to weak stimulation and overreactivity are independent traits.

### Perceptual Sensitivity and Overreactivity As Independent Traits

To test the unidimensionality of the HSP scale Evans and Rothbart (2008) carried out factor analysis on the HSP scores taken from a sample of 297 undergraduates and compared the factor scores with scores on several scales of the Adult Temperament Questionnaire (ATQ; Evans and Rothbart, 2007). The ATQ is based on a multidimensional approach to temperament that subdivides each central temperamental trait into several sub-traits. It enables fine-grained exploration of relationships between these traits (Derryberry and Rothbart, 1988). Importantly, the questionnaire includes separate scales for assessing sensitivity to low-intensity stimulation and perceptual discomfort (overreactivity) due to high-intensity stimulation. Definitions of these ATQ (sub)scales can be found in **Table 1**.

**Table 1:** Overview of the main constructs and operational definitions based on the Adult Temperament Questionnaire used in the current study.

Label of construct in the current study	ATQ (sub)scale used to measure the construct, definition of the (sub)scale, and sample item
**Sensitivity** (for weak stimuli)	**Orienting sensitivity** Tendency for “Automatic attention to both external sensory events and internal events”^a^ Includes the subscale**Neutral perceptual Sensitivity** Tendency for “Awareness of slight, low intensity stimulation arising from the external or internal environment. *I often notice visual details in the environment”*^b^
**Punishment reactivity** (to strong stimuli) Also: reactive avoidance to strong stimuli, overreactivity, or low tolerance.	**Discomfort**Tendency to experience “Unpleasant affect resulting from the sensory qualities of stimulation. *I find loud noises to be very irritating”*^b^
**Reward reactivity** (to strong stimuli) Also: reactive approach to strong stimuli	**High intensity pleasure** Tendency to experience “Pleasure related to situations involving high stimulus intensity, rate, complexity, novelty, and incongruity. *I would not enjoy the sensation of listening to loud music with a laser light show* (coded in reverse)”^b^

*^a^Evans and Rothbart (2008, p. 111).*

*^b^Evans and Rothbart (2007, Appendix A, p. 884–885).*

Factor analysis on the HSP items indicated that the HSP scale consisted of two separate factors. The first factor was strongly associated with ATQ negative affectivity and its discomfort subscale in particular. The other factor correlated highly with ATQ orienting sensitivity and its neutral perceptual sensitivity subscale (Evans and Rothbart, 2008). These results do not support the unidimensionality of the HSP scale. Furthermore Evans and Rothbart (2008) did not find a relationship between neutral perceptual sensitivity and discomfort, between orienting sensitivity and discomfort, or between the two factors of the HSP scale. The absence of these relationships questions the unidimensional view that individuals with high sensitivity to weak stimuli also have high reactivity to strong stimulation.

Other findings that conflict with the unidimensional view were reported by Ellermeier et al. (2001). In a group of 61 volunteers they investigated the idea that increased reactivity to noise in the environment is (partly) due to increased auditory acuity. To measure reactivity to noise they used a psychometrically evaluated noise sensitivity questionnaire. Note that the term noise sensitivity in this study referred to a stable personality trait concerning perceptual, cognitive, affective and behavioral reactivity toward environmental noises. Noise sensitivity as measured by this questionnaire does thus not refer to sensitivity to weak stimuli, but can be regarded as measure of reactivity (or discomfort in terms of the ATQ) in the auditory domain. Auditory acuity was measured using several measures, including an adaptive forced-choice measure of the absolute threshold of hearing, which can be regarded an objective psychophysical measure of sensitivity to low intensity stimulation. This measure is of specific interest here because it is similar, although methodologically improved, compared to the measures used in the empirical studies discussed above (Smith, 1968; Siddle et al., 1969; Edman et al., 1979) that found relations between perceptual sensitivity and extraversion. Ellermeier et al. (2001), however, found no significant relationship between their measure of reactivity to noise and auditory acuity, including the threshold of hearing. In line with the conclusions of Evans and Rothbart (2008), this finding suggests that reactivity and sensitivity, at least in the auditory domain, are independent from each other and are not originating from a single trait.

Taken together, there is some evidence supporting the claim that sensitivity to weak stimuli and overreactivity to strong stimuli arise from the same trait (Smith, 1968; Siddle et al., 1969; Edman et al., 1979). However, other research findings suggest that sensitivity to weak stimuli and overreactivity are independent traits (Ellermeier et al., 2001; Evans and Rothbart, 2008). There is thus disagreement about the relationship between sensitivity to weak stimuli and overreactivity to strong stimuli. A solution may be found in PARCS theory (Tops et al., 2010, 2014). In the next paragraphs we will briefly set out PARCS theory and its predictions regarding the relationship between sensitivity to weak stimuli and overreactivity to strong stimuli. Because in the present study we operationalized constructs of PARCS theory using ATQ scales (Evans and Rothbart, 2007), we will relate these constructs to the labels used in the adult temperament model by Derryberry and Rothbart (1988), Evans and Rothbart (2007) when we introduce them.

### Perceptual Sensitivity and Overreactivity in PARCS Theory

Predictive and Reactive Control Systems theory provides an integrative framework for understanding psychological states and traits based on functioning of two major control systems in the brain: the predictive and reactive control system. These systems regulate cognition, autonomic responses, behavior, homeostasis, and emotion. PARCS theory describes temperamental or personality traits as dispositional bias toward the reactive or toward the predictive control systems, which are each adaptive in specific environments and contexts. Predictive temperament, which we will also refer to as high predictivity, is characterized by dispositional bias toward the predictive system. The predictive system controls behavior based on internal models that predict which actions will be effective for reaching goals in a given context and allows planning for future events. Predictive temperaments likely evolved to be adaptive in predictable environments, and predictive control is still deemed adaptive in such environments in modern day life (Tops et al., 2010, 2014). For example, when driving in a familiar city with organized traffic where traffic rules are obeyed one can adopt a largely feedforward approach, following previously learned rules and habits applicable to the current context, and plan behavior based on predictions about the future (such as planning ahead what is the best route to arrive home on time while passing by the cheapest gas station and do the weekly groceries on the way). In such a situation a predictive temperament is thus advantageous. In contrast to predictive temperaments, reactive temperaments are characterized by dispositional bias toward the reactive systems, which control behavior in a momentary fashion through feedback from the continuous stream of external stimuli. Reactive control is adaptive in novel, unpredictable and unstable environments (Tops et al., 2010, 2014). For example when driving for the first time in a foreign city with disorganized, busy traffic where other drivers do not (seem to) comply with traffic rules, one needs to adopt a feedback guided strategy, be constantly vigilant to environmental stimuli, and ready to immediately respond to the rapidly and unexpectedly changing situational demands (such as a car ending up in front of you after suddenly changing multiple lanes). In this situation a reactive temperament, which we will also refer to as high reactivity, is thus more suitable than a predictive temperament.

Crucial to the current study, PARCS theory distinguishes two types of reactive systems: the reactive avoidance system and the reactive approach system. In line with other biopsychological theories of personality and temperament based on research in humans (e.g., Cloninger et al., 1993; Corr, 2004; Evans and Rothbart, 2007; cf. Gray and MacNaughton, 2000), the discrimination of the reactive approach and avoidance systems is inspired on the model of anticorrelated reward- and punishment systems developed by Gray (1970, 1989) on the basis of animal research. Individuals with bias toward the reactive avoidance system have a strong drive to process (potentially) aversive stimuli and experience aversion in order to avoid these stimuli. This drive is expressed as elevated anxiety and harm avoidance (Pickering and Gray, 1999). We will refer to dispositional bias toward the reactive avoidance system as high punishment reactivity. In the adult temperament model developed by Derryberry and Rothbart (1988), Evans and Rothbart (2007) this construct is labeled as negative affect. In addition to fear, sadness and frustration it encompasses discomfort, which, as discussed above reflects aversive responding to strong stimuli (Derryberry and Rothbart, 1988; Evans and Rothbart, 2007). Discomfort can thus be understood as punishment reactivity specifically toward strong sensory stimulation. On the other hand, individuals with bias toward the reactive approach system have a strong drive to process (potentially) appetitive stimuli in order to approach these stimuli. This drive is expressed as elevated reward responsiveness and sensation seeking (Pickering and Gray, 1999). We will refer to dispositional bias toward the reactive approach system as high reward reactivity. This construct is labeled extraversion/surgency in Derryberry and Rothbart (1988), Evans and Rothbart (2007) temperament model. Besides sociability and positive affect it includes high intensity pleasure, which reflects the tendency to derive pleasure from strong stimuli (Derryberry and Rothbart, 1988; Evans and Rothbart, 2007). High intensity pleasure can thus be regarded as reward reactivity specifically toward strong sensory stimulation. According to PARCS theory, depending on the dispositional bias toward the reactive approach or avoidance system one has, the same stimulus may be experienced differently. When confronted with strong stimuli such as loud music, individuals with high punishment reactivity will have a tendency to experience these stimuli as aversive and to be avoided, i.e., as punishment. By contrast, individuals with high reward reactivity will have a tendency to experience these stimuli as pleasurable and to be approached, i.e., as reward.

Predictive and Reactive Control Systems theory builds on, and somewhat reorganizes the above theories of reactive approach and avoidance systems, additionally based on evidence from brain lesion and neuroimaging studies in humans (Tops et al., 2010, 2014, 2016). Furthermore, as mentioned earlier, PARCS theory adds a predictive system to the model. In contrast to the reactive system that enables immediate, mutually incompatible avoidance and approach responding to novel, urgent punishment and reward stimuli, the predictive system utilizes internal models of effective ways to respond to familiar stimuli and contexts. Those internal models represent relationships between entities, motivations, actions, and outcomes and are formed by prior learning during exposure to similar stimuli (Quirin et al., 2015). When the individual encounters similar situations in the future, integrated experiences stored in the internal model can be recalled and will provide context and perspectives for perception and appraisal of the situation and potential actions. Note that this can also apply to punishments or rewards that have been previously integrated into internal models. When presented with a previously integrated compared to a novel punishment or reward stimulus an individual can more readily and flexibly switch from reactive control, with its narrow focus on the salient stimulus, to predictive control, which is less emotionally reactive and more mindful and provident in nature (Tops et al., 2014; Quirin et al., 2015).

Although the reactive system is specifically equipped for immediate responding to stimuli in rapidly changing environments, this does not mean that it operates without higher levels of cognitive processing. According to PARCS theory both the predictive and the reactive system have attentional and cognitive control functionality, but each has a different mode of processing. Reactive control is feedback guided and includes processes such as orienting, appraisal (i.e., assessment of stimuli in the environment on significance for well-being; Moors et al., 2013), working memory maintenance, and actively sustained attention such as needed for detection of infrequent stimuli. Predictive control, on the other hand, works in a feed-forward fashion, including processes such as planning for future events and inductive reasoning (Tops et al., 2014). This distinction in reactive and predictive cognitive processes is also reflected in neuroimaging and anatomical data. Cortical areas of the reactive system that regulate reactive reward and punishment systems (Tops et al., 2014), such as the anterior insula (AI) and dorsal anterior cingulate cortex (dACC), receive many projections from limbic and subcortical areas of the reactive punishment and reward systems such as the amygdala and ventral striatum. By contrast, cortical areas of the predictive control system, such as the posterior cingulate cortex and precuneus, receive less such projections but seem to downregulate those areas (Devinsky et al., 1995; Tops and Boksem, 2012).

**FIGURE 1:**
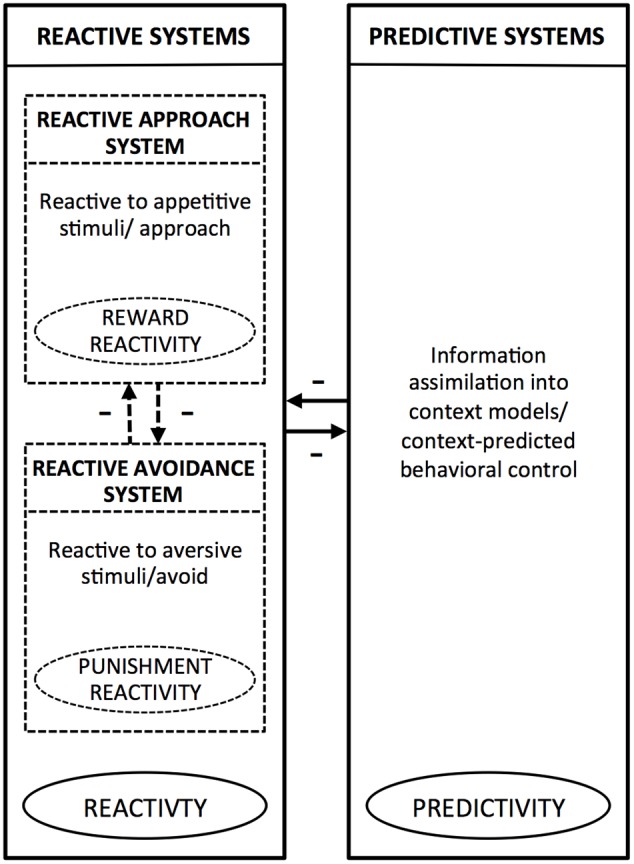
Schematic overview of the reactive (approach and avoidance) and predictive systems according to the Predictive and Reactive Control Systems (PARCS) theory. Within the boxes, which represent the systems, we provide a description of characteristic information processing/behavior mediated by the given system. The encircled terms indicate the temperamental tendencies that arise from bias toward the given system. The arrows indicate inhibitory relationships between the reactive systems and predictive systems, and between the reactive approach system and reactive avoidance system.

Integrating the above and other evidence, PARCS theory (Tops et al., 2010, 2014, 2016) suggests that the reactive system and the predictive system tend to inhibit each other, producing anticorrelated activation. At the same time, within the reactive system, the approach and avoidance systems also tend to inhibit each other (Gray, 1970, 1989). Accordingly, reward and punishment reactivity have in common that both reflect reactive, rather than predictive, temperaments, as both are mediated by reactive systems. At the same time these temperaments oppose each other because, in immediate, reactive action control, each reflects a different action orientation (approach or avoid). Thus, PARCS theory predicts that both reward and punishment reactivity are positively related to reactivity and negatively related to predictivity. It also predicts that reward and punishment reactivity are negatively related to each other. **Figure 1** shows how, according to PARCS theory, punishment reactivity and reward reactivity relate to each other, and how both of these temperamental tendencies relate to predictivity. In the next paragraphs, we will further argue how this framework may help to elucidate the relation between sensitivity to weak stimuli and overreactivity (punishment reactivity) to strong stimuli.

The present empirical paper does not provide the space to review all evidence behind PARCS theory (for this we refer to review papers, e.g., Tops et al., 2010, 2014, 2016), however, we provide some examples from neuroimaging research that show integrated responding to both aversive and appetitive stimuli in areas that also facilitate processing of weak or ambiguous stimuli. Cortical components of the reactive system in PARCS (e.g., the AI and dACC) match what has been named the “salience network” in human neuroimaging studies: a key network in sensory perception and attention allocation (Seeley et al., 2007). Besides receiving projections from networks that seem more strongly involved in either reward or punishment processing, the salience network responds to salient stimuli in general, both appetitive and aversive (Hayes and Northoff, 2012; Hayes et al., 2014). Moreover, higher connectivity within the salience network was found to be associated with higher individual differences scores of harm avoidance and anxiety (Markett et al., 2013) and decreased connectivity of this network with areas of the reward system was related to decreased extraversion in depressed patients (van Tol et al., 2013). At the same time, the salience network seems involved in processing stimulus salience or relevance to a current task (e.g., detecting a sound) and to be activated whenever sensory input poses a challenge by sensory uncertainty or ambiguity, the disambiguation of which requires enhanced effort and alertness (Sterzer and Kleinschmidt, 2010; Lamichhane and Dhamala, 2015).

The evidence from neuroimaging studies for involvement of the salience network in processing of aversive and appetitive stimuli as well as weak or ambiguous stimuli converges with temperament and personality research. In terms of personality, PARCS theory suggests that trait absorption reflects individual inclinations toward salience network activation (Tops et al., 2016). Absorption is defined as the tendency to get attentionally immersed in and elaborately appraise salient sensory or emotional (positive and negative) experiences and one’s internal state (Gohm and Clore, 2000; Tops et al., 2016) and as such corresponds to the attentional functions of the reactive system, which include orienting responses and appraisal of salient stimuli. The notion that absorption is a correlate of the salience network in the reactive system is supported by findings that activation of areas in the salience network showed correlation with trait absorption (Tops and Boksem, 2010) and state absorption (Wilson-Mendenhall et al., 2013; Hsu et al., 2014). It is also in line with findings that participants scoring high on absorption showed enhanced processing of emotionally neutral task relevant stimulus features as well as enhanced processing of task irrelevant emotional features compared to participants scoring low on absorption. This was reflected in reaction times (RTs) as well as in event related brain potentials (ERPs) to a task in which participants determined whether the letter A, the task-relevant stimulus feature, was present in a word or not. Participants scoring high on absorption responded faster to words with the letter A than without the letter A, while low absorption participants did not show such RT difference. High absorption participants also showed an increased sustained widespread positivity to words containing the letter A, labeled as Late Positive Complex (LPC), compared to low absorption participants, indicating enhanced processing of task relevant features. Furthermore, for high absorption participants only, RT was further decreased and the LPC was further increased when the A occurred in a word with emotional compared to emotionally neutral meaning, indicating that processing of task-irrelevant emotional features was also enhanced for these participants (de Ruiter et al., 2003, 2006). Absorption can be measured on various scales (Tops et al., 2016) including the Openness to Experience subscale of personality inventories based on the five factor model of personality (McCrae and Costa, 1987, 1997; Costa and McCrae, 1992). This is supported by studies finding large correlations between openness to experience (especially its fantasy, aesthetics and feelings facets) and other absorption scales, such as the Tellegen Absorption Scale (Glisky et al., 1991; McCrae, 1993). Absorption and openness to experience conceptually also strongly overlap with orienting sensitivity in Evans and Rothbart (2007) temperament model, which is supported by large correlations found between openness to experience and ATQ orienting sensitivity (Wiltink et al., 2006). In the current study we will therefore use ATQ orienting sensitivity as measure of perceptual and attentional aspects of the reactive system. In comparison to other measures of absorption, the ATQ orienting sensitivity scale is particularly suitable for the present study, because its subscales uniquely focus on orienting to and appraising of weak and subtle stimuli (Evans and Rothbart, 2008). This scale thus provides a measure of sensitivity to weak stimuli that reflects perceptual and attentional aspects of reactivity in PARCS theory.

As reviewed above, both the mutually anticorrelated approach (reward) and avoidance (punishment) systems input to, and activate, the cortical areas of the reactive control system. In turn, the activation of reactive control increases the allocation of attentional resources to aversive and appetitive stimuli, as well as to relevant weak stimuli, which is supported by findings on the perceptual and attentional correlates of trait absorption (de Ruiter et al., 2003, 2006) and the perceptual and attentional correlates of salience network activation (Sterzer and Kleinschmidt, 2010; Hayes and Northoff, 2012; Hayes et al., 2014; Lamichhane and Dhamala, 2015). We therefore expect that orienting sensitivity, as measure of perceptual and attentional aspects of reactivity, positively associates to other measures of sensitivity to weak stimuli, such as sensory detection thresholds. We also expect that orienting sensitivity is related to both the mutually anticorrelated traits of discomfort (punishment reactivity to strong stimuli) and high intensity pleasure (reward reactivity to strong stimuli). In the next paragraph, we will argue more specifically, based on PARCS theory, how taking into account both punishment reactivity and reward reactivity may help to understand the relation between sensitivity to weak stimuli and overreactivity (punishment reactivity) to strong stimuli.

As argued above, based on PARCS theory we predict that perceptual sensitivity is related to both punishment and reward reactivity. However, because the two types of reactivity are also negatively related to each other, they are possible suppressor variables that may cancel out the separate positive relations between each reactivity measure and perceptual sensitivity (MacKinnon et al., 2000). We will illustrate the idea of (statistical) suppression with an example in the auditory domain because the current study included a measure of sensitivity in the auditory domain. PARCS theory predicts that sensitivity to weak sounds, given its association with reactivity, is high in individuals who have a tendency to experience aversion (high punishment reactivity) when exposed to noise or loud sounds and also in individuals who have a tendency to experience pleasure (high reward reactivity) when exposed to noise or loud sounds. At the same time, the tendency to experience aversion due to noise or loud sound is inversely related to the tendency to derive pleasure from it: the more one tends to experience aversion from intense sound the less one tends to experience pleasure from it. Now, take an individual who has a very low tendency to experience aversion from strong sound. On the one hand this person is expected to score relatively low on sensitivity to weak sounds. On the other hand, however, this person is also likely to experience strong pleasure from loud sound and therefore is actually likely to score high on sensitivity. If this is the case, extreme scores on both ends of a scale measuring the tendency to experience aversion to strong sounds are associated with high sensitivity. Therefore, across individuals, no positive relationship between deriving displeasure and sensitivity will be observed. This example illustrates that the positive relation between punishment reactivity and perceptual sensitivity can be canceled out due to the negative relation between punishment reactivity and reward reactivity (and vice versa). This type of statistical relationship is known as a suppression effect or inconsistent mediation (MacKinnon et al., 2000). If these suppression effects occur, both types of reactivity will show to be related to sensitivity when controlled for each other, but this relationship is canceled or dampened when not controlled for each other. Thus, according to PARCS theory, in order to gain proper understanding of the relation between sensitivity to weak stimuli and reactivity to strong stimuli, it is crucial to take the suppression effects into account. In the current study we tested the predicted suppression effects.

### Current Study

As discussed above, in the current study we used (sub)scales of the ATQ to measure punishment and reward reactivity to strong stimuli and sensitivity to weak stimuli, thereby building on the work of Evans and Rothbart (2007, 2008). To summarize, the ATQ discomfort scale measured punishment reactivity to strong stimuli, the ATQ high intensity pleasure (HIP) scale measured reward reactivity to strong stimuli, and the ATQ orienting sensitivity scale measured sensitivity to weak stimuli. **Table 1** provides definitions and sample items of these ATQ scales. To test the predicted suppression effects we performed a correlational analysis on the ATQ scores. We expected to find a relation between ATQ discomfort and ATQ orienting sensitivity when ATQ HIP would be added as control variable to the analysis, but a weaker, or absent, relation when ATQ HIP would not be taken into account. Similarly, we expected to find a relation between ATQ HIP and ATQ orienting sensitivity when ATQ discomfort would be added as control variable to the analysis, but a weaker or absent relationship when ATQ discomfort would not be taken into account.

**FIGURE 2:**
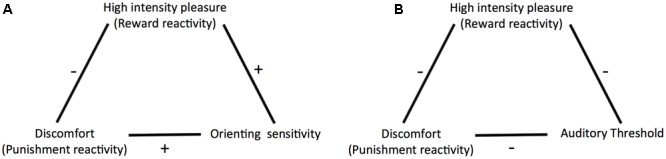
Relationships as predicted by Predictive and Reactive Control Systems (PARCS) theory and tested in the current study **(A)** between discomfort (punishment reactivity), high intensity pleasure (reward reactivity), and orienting sensitivity, and **(B)** between discomfort, high intensity pleasure, and auditory threshold.

Further, building on previous studies investigating the relationship between sensitivity to weak stimuli and traits associated with overreactivity to strong stimuli (Smith, 1968; Siddle et al., 1969; Edman et al., 1979; Ellermeier et al., 2001) we included a psychophysical measure of perceptual sensitivity in addition to ATQ orienting sensitivity. We chose a measure in the auditory domain because previous studies in this domain yielded mixed conclusions regarding the question whether sensitivity and reactivity arise from the same trait. The inclusion of an objective psychophysical measure is also important because ATQ orienting sensitivity is a rating scale measure of sensitivity to weak stimuli. Rating scale measures may be prone to response bias, which is a “systematic tendency to respond to questionnaire items on some basis other than the specific item content” (Paulhus, 1991, p. 17). There are various types of response biases including social desirability bias, where participants answer in such a way that they give a good impression of themselves regardless of their true characteristics (Furnham and Henderson, 1982; Paulhus, 1991), and extremity bias which is the tendency to give extreme rather than moderate responses (or vice versa) irrespective of the content of the items (Bachman and O’Malley, 1984; Paulhus, 1991; Mõttus et al., 2012). Response bias can impact the magnitude of the means and standard deviations of single scales as well as correlations between scales (Baumgartner and Steenkamp, 2001; Van Vaerenbergh and Thomas, 2013). Including an objective psychophysical measure of sensitivity enabled us to check whether our results could be explained by response bias or not. We used the masked auditory detection threshold for pure tones, which reflects listeners’ ability to detect faint sounds in noise, as an objective indicator of sensitivity. To measure it we used a two-interval forced choice (2IFC) procedure combined with a staircase procedure (García-Pérez, 1998). This procedure is regarded as criterion-free, that is, it measures sensitivity irrespective of the response criterion used by the observer (Green and Swets, 1966; Kingdom and Prins, 2010) and thus has minimized vulnerability to effects of response bias. If self-reported sensitivity truly reflects perceptual sensitivity, it should correlate similarly to the reactivity scales as objectively measured sensitivity does. **Figure 2** shows an overview of the relationships we aimed to test in the current study.

## Materials and Methods

### Participants

Eighty-one participants (Age: *M* = 20.5, *SD* = 2.0, 18–27 years; 20 males) with no self-reported hearing problems or depression took part either for course credit or payment. Data from three participants were not included in the analyses because they had strongly deviating thresholds (above the three inter quartile range criterion). The present study was part of a larger investigation with the same participants on affective and temperamental influences on the masked auditory threshold. Part of this investigation contributed to a study on modulation of the masked auditory threshold by mood state (Bolders et al., 2017). In the current study only the threshold measured prior to the mood induction was used to examine its relationships with individual differences in temperament.

### Apparatus

Sound was binaurally presented through insert earphones (Etymotic ER-4B microPro). These earphones provide 35 dB external noise attenuation. Stimulus presentation was controlled by E-prime 2 (Schneider et al., 2002) using a computer with a CRT screen (75 Hz refresh rate, 1024 × 768 resolution). Responses were made on a QWERTY keyboard and by using a mouse.

### Questionnaire

Temperament was assessed using the short version of the ATQ translated into Dutch by Hartman and Majdandžić (2001). This questionnaire consists of 77 items and contains the same constructs and sub constructs as the original ATQ (Evans and Rothbart, 2007). Each item is formulated as a statement. Participants in the current study were asked to indicate how applicable this statement was to them by clicking on the appropriate answer option presented on the computer screen. We used a 6-point scale varying from “Not at all” (1) to “Completely” (6). There was also a “not applicable option,” which was treated as missing data point. Supplementary Table 3 shows the number of items and Cronbach’s alpha per (sub)scale.

### Threshold Task

#### Sounds

Two wav files were created with Audacity software, one to serve as a signal and one as non-signal in the threshold task. The signal was a 1 kHz pure tone, 500 ms in duration with 10 ms ramped on- and offset. An empty sound file of 500 ms served as non-signal. A white noise (20–10 kHz band-filtered) generated with Goldwave software was used as masking noise that was constantly present during the threshold task. Sound levels at output were calculated from the voltages delivered at the earphone input measured with an oscilloscope (Tektronix TDS2002) and the earphone efficiency reported by the earphone manufacturer (108 dB SPL for 1Vrms in a Zwislocki coupler, ER-4 datasheet, Etymotic Research, 1992). The white noise was presented with a voltage delivered at the earphone input that would equal 38 dB SPL output for a 1 kHz tone. Digital sound properties for all sounds were standardized (44 kHz, 16 bit, mono).

#### Task Procedure

An adaptive two interval forced (2IFC) choice task was employed to measure the auditory threshold. At the beginning of each trial a fixation cross was presented in the center of the screen for 1000 ms. This was followed by two observation-intervals which were marked with a number (1 or 2) presented in the center of the screen. The intervals were separated by a blank interval during which only a fixation cross was presented. The three intervals were each 700 ms in duration. On every trial one of the two observation-intervals was pseudo-randomly selected to contain the signal with the constraint that no more than four trials with the same selected interval could occur in succession. The 500 ms signal was centered in the 700 ms observation interval. After the second observation interval there was a 100 ms blank screen. This was followed by a red “X” presented in the center of the screen until the participants indicated whether they had heard the signal in the first or the second interval by pressing the z-key on the keyboard with their left index finger or the m-key on the keyboard with their right index finger, respectively. The sound level of the signals depended on the performance of the participants and increased or decreased adaptively according to a transformed and weighted up/down rule (García-Pérez, 1998). A 1-up/2-down rule was used and the ratio of the step size down and step size up was 0.548. In other words, after one incorrect trial the sound level of the tone went up one step (e.g., 3 dB), but it went down one step only after two consecutive correct trials, with the step size up being 1.82 times the size of the step down. This rule has been shown to reliably converge to 80.35% correct performance (García-Pérez, 1998). Initially the step size down was 15 dB. This changed to 5 dB after two reversal points (trials at which the sound level changed from going up to down or vice versa) and to 3 dB after four more reversal points. The initial sound level was 68 dB SPL. To calculate the threshold (sound level needed for a performance of 80.35% correct) the sound levels of tones at the last ten reversal points were averaged. The e-prime script for the adaptive procedure was adapted from Hairston and Maldjian (2009).

### Experiment Procedure

After providing informed consent, participants were seated in a comfortable chair at 50 cm from the computer monitor in a quiet dimly lit individual test cubicle. They were instructed about the flow of the experiment, practiced with correct earphone insertion and the experimenter verified whether external sounds were indeed attenuated. Further instructions followed on the computer screen. Regarding the threshold task it was explained that the signal would be presented equally often in each interval, and that, although the signal could be difficult to hear on some trials, it was important to keep paying attention to the task and that an answer was required on all trials. The task instructions stressed accuracy and all responses were self-paced. Participants carried out eight practice trials in order to get used to the task. The practice trials were equal to the trials of the threshold task, except that the sound level of the signals was kept at 68 dB SPL and after each practice trial participants received feedback about their accuracy. Following the practice trials the threshold task started. At the end of the study participants filled out the ATQ.

### Data Analyses

#### Validation Analyses

To validate the self-report measure of sensitivity, we correlated the threshold with ATQ orienting sensitivity. ATQ orienting sensitivity includes perceptual sensitivity as a subscale (five items), but also includes subscales measuring sensitivity to experiencing divergent mental associations or images (five items) and sensitivity to subtle affective stimuli (five items). Because ATQ orienting sensitivity is a more reliable (cronbach’s α = 0.67 in current study) measure of sensitivity to subtle stimuli than each of its subscales separately (see Supplementary Table 3 for cronbach’s αs), we used the orienting sensitivity scale in subsequent analyses as self-report measure of sensitivity. It is worth mentioning here that the threshold correlated similarly with each ATQ orienting sensitivity subscale (see Supplementary Table 2 for correlation coefficients).

#### Main Analyses

To answer our main question about the relation between sensitivity to weak stimuli and reactivity to strong stimuli, we examined the partial correlations between ATQ orienting sensitivity and the perceptual reactivity scales (ATQ discomfort and ATQ HIP). The correlation between ATQ orienting sensitivity and ATQ discomfort was controlled for ATQ HIP and the correlation between ATQ orienting sensitivity and ATQ HIP was controlled for ATQ discomfort. We carried out the same analyses replacing ATQ orienting sensitivity with the masked auditory threshold.

To examine the expected suppression effects we repeated the above described correlational analyses but without controlling for ATQ HIP or ATQ discomfort. If ATQ HIP and ATQ discomfort suppress each other’s association with perceptual threshold and sensitivity, then not controlling for the suppressing variable should substantially reduce the correlations.

In all of the main analyses several other variables were controlled for. First, because we were interested in the relationships with the perceptual aspects of punishment and reward reactivity, we controlled for the broader constructs of ATQ frustration and ATQ positive affect. Where ATQ discomfort measures irritability due to intense stimulation (e.g., “I find loud noises to be very irritating”), ATQ frustration measures irritability in general (e.g., “It doesn’t take very much to make me feel frustrated or irritated”). And, where ATQ HIP measures the tendency to experience pleasure due to intense stimuli (e.g., “I would enjoy the sensation of listening to loud music with a laser light show”), ATQ Positive Affect measures the tendency to experience pleasure in general (e.g., “It doesn’t take much to evoke a happy response in me”). We controlled for ATQ frustration because people who indicate on the ATQ discomfort scale that they get irritated from intense stimuli may actually get irritated easily in general, not only by intense stimuli. Similarly, we controlled for ATQ positive affect because people who indicate on the ATQ HIP scale that they derive pleasure from intense stimuli may actually tend to derive pleasure from things more in general, not specifically from intense perceptual stimuli.

Second, we also controlled for sex because this variable has been associated with discomfort and HIP (Ormel et al., 2005) and hearing sensitivity (Robinson, 1988). Age has also been associated with discomfort or unpleasantness experienced due to high arousal stimuli (Keil and Freund, 2009; Tops and Matsumoto, 2011) and with hearing sensitivity (Robinson, 1988). However, the age range was small and adding age as a control variable did not affect the pattern of the correlations. Therefore the analyses are presented without controlling for this variable.

## Results

### Validation Analyses

To validate the self-report measure of sensitivity, we correlated the threshold (*M* = 21.14 dB SPL, *SD* = 1.93) with ATQ orienting sensitivity. The threshold had a moderate negative relationship with orienting sensitivity, *r* = -0.31, *p* = 0.006. The Supplementary Material provides a full matrix of the uncorrected correlations between all ATQ scales (Supplementary Table 1) and subscales (Supplementary Table 2) and the threshold. It also provides a table with descriptive statistics for the ATQ (sub)scales (Supplementary Table 3).

### Main Analyses

**FIGURE 3:**
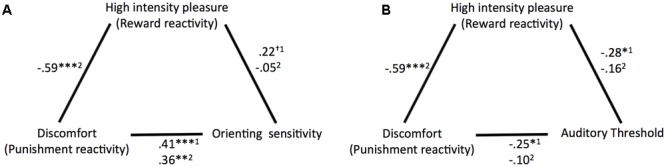
Overview of the partial correlation coefficients and significance levels of the relationships **(A)** between discomfort (punishment reactivity), high intensity pleasure (reward reactivity), and orienting sensitivity and **(B)** between discomfort, high intensity pleasure, and auditory threshold. ^1^Controlled for sex, frustration, positive affect, and HIP or discomfort. ^2^Controlled for sex, frustration, positive affect. ^†^*p* < 0.10, ^∗^*p* < 0.05, ^∗∗^*p* < 0.01, ^∗∗∗^*p* < 0.001.

Correlation coefficients and significance levels of the relationships tested for the main analyses are provided in **Figure 3**. ATQ orienting sensitivity displayed significant partial correlations with ATQ discomfort and trends of similar magnitude with ATQ HIP when ATQ discomfort and ATQ HIP were controlled for each other and for ATQ frustration, ATQ positive affect, and sex. Similarly, the masked auditory threshold displayed significant partial correlations with ATQ discomfort and with ATQ HIP, when ATQ discomfort and ATQ HIP were controlled for each other and for ATQ frustration, ATQ positive affect, and sex.

When ATQ discomfort and ATQ HIP were not controlled for each other, most correlations between the (self-reported and objective) sensitivity and self-reported reactivity measures were low and not significant. Only ATQ orienting sensitivity still correlated with ATQ discomfort, albeit with slightly lower magnitude.

## Discussion

The present study examined the relations between self-reported and objectively measured sensitivity to subtle stimuli and self-reported reactivity to strong stimuli within the framework of PARCS theory. Importantly, two types of reactivity are distinguished in this theory: punishment reactivity and reward reactivity. We measured punishment reactivity to strong stimulation by means of the ATQ discomfort scale and reward reactivity to strong stimulation with the ATQ HIP scale. Sensitivity to weak stimuli was measured using the objectively determined masked auditory threshold as well as the ATQ orienting sensitivity scale, which is a self-report measure of sensitivity to weak stimuli that reflects perceptual and attentional aspects of reactivity in PARCS theory.

As predicted from PARCS theory, our results showed that ATQ orienting sensitivity was positively associated with objectively determined sensitivity (inverse of the masked auditory threshold). Furthermore, and crucial for answering our research question, both types of reactivity to strong stimulation related to (self-reported and objectively measured) sensitivity to weak stimuli, but only when controlled for each other, indicating a mutual suppression effect. These findings are in line with the notion of PARCS theory that punishment and reward reactivity overlap in terms of reactivity toward stimulation, but that these tendencies also oppose each other in terms of response orientation (approach or avoid). Note that associations between the reactivity measures and self-reported sensitivity to weak stimuli were replicated when sensitivity was objectively measured as the masked auditory threshold, which makes it unlikely that the associations between sensitivity to weak stimuli and the reactivity to strong stimuli were driven by response bias.

Our results may help to understand previous inconsistencies in the literature with respect to the dependency of overreactivity to strong stimuli and perceptual sensitivity to weak stimuli. Some studies found support for the claim that sensitivity to weak stimuli and overreactivity arise from the same trait (Smith, 1968; Siddle et al., 1969; Edman et al., 1979), while other research findings suggested that sensitivity to weak stimuli and overreactivity are independent traits (Ellermeier et al., 2001; Evans and Rothbart, 2008). Our study demonstrated that when not controlled for reward reactivity to strong stimuli, the relation between punishment reactivity to strong stimuli and perceptual sensitivity might be suppressed. If this is the case, the dependency between punishment reactivity and sensitivity becomes apparent only when reward reactivity is kept constant. Discrepant and zero findings can be expected because differences in the distributions of reward reactivity introduce differences in the relation between punishment reactivity to strong stimuli and sensitivity to weak stimuli. The same holds for the relation between reward reactivity to strong stimuli and sensitivity to weak stimuli when not controlled for punishment reactivity. As previous studies did not take both punishment and reward reactivity to strong stimuli into account, this might explain their discrepant findings regarding the dependency of overreactivity to strong stimuli and perceptual sensitivity to weak stimuli.

Because Eysenck’s (1967) personality theory is such a well-known and influential theory that considers sensitivity to weak stimuli and overreactivity to strong stimuli as resulting from one underlying trait, we will specifically compare our results to this theory. Our findings are in line with the ideas of Eysenck in the sense that overreactivity to strong stimuli and sensitivity to weak stimuli seem to be dependent. The pattern of dependencies we found, however, does not agree with Eysenck’s predictions, which are based on introversion-extraversion as underlying trait. According to Eysenck, overreactivity to high intensity stimulation is associated with high sensitivity to weak stimulation (Eysenck, 1967). This does match our finding that punishment reactivity to strong stimuli, when controlled reward reactivity, was associated with sensitivity to weak stimulation. Eysenck (1967), however, also suggested that extraverts’ enjoyment of high intensity stimulation is associated with low sensitivity to weak stimulation. By contrast, we found that, when controlled for punishment reactivity to strong stimulation, pleasure from high intensity stimulation was associated with high sensitivity to weak stimulation. This renders it unlikely that sensitivity to weak stimuli and overreactivity are associated due to introversion-extraversion as underlying trait. Instead, as suggested by PARCS theory the pattern of dependencies can be explained by individual differences in the tendency to activate the salience network of the reactive system. This network mediates processing of aversive and appetitive stimuli as well as relevant weak stimuli, and receives input from both the mutually anticorrelated approach (reward) and avoidance (punishment) reactive systems. Furthermore, as discussed above, our findings stress the importance of taking into account both reward and punishment reactivity to understand the relationship between sensitivity to weak and overreactivity to strong stimuli. This favors the use of these two dimensions, which are based on Gray’s early conceptions of personality (Gray, 1970, 1989) over Eysenck’s introversion–extraversion dimension in studying the relation between sensitivity and overreactivity. Moreover, Gray’s (1970, 1989) dimensions not only fit better with the current results, but also seem to better account for earlier findings. According to Gray (1970, 1989) punishment reactivity (which he labeled anxiety) is, in terms of Eysenck’s personality dimensions, reflected in a combination of high introversion and high neuroticism. Relating sensitivity to punishment reactivity rather than to introversion thus seems to better account for findings of confounding and interaction effects by neuroticism in earlier studies that investigated the relation between introversion and the threshold for noticing weak stimuli (Siddle et al., 1969; Edman et al., 1979).

In addition to furthering the understanding of the relation between sensitivity to weak and reactivity to strong stimuli, our findings also have relevant implications for the study of temperamental and psychophysical determinants of noise annoyance or annoyance produced by other environmental nuisances in daily life. This regards, for example, the question whether noise sensitivity, a measure of discomfort in the auditory domain that predicts noise annoyance, is dependent on basic auditory perception or not (Ellermeier et al., 2001). As our results demonstrated, perceptual reward reactivity (HIP) may suppress the relation between auditory punishment reactivity (discomfort) and perceptual sensitivity. Therefore, in order to gain a more complete picture of the determinants of noise annoyance, we recommend including measures of reward reactivity to strong stimuli in future studies and controlling for it. In addition to noise annoyance, PARCS theory might also provide a framework to contribute to the understanding of other environmental intolerances, such as multiple chemical sensitivity (MCS). MCS, also known as idiopathic environmental intolerance (IEI), is a condition that is characterized by intolerance for chemical agents expressed as various somatic complaints including fatigue, headaches and pain (Graveling et al., 1999; Bornschein et al., 2002). Interestingly, MCS has been associated with anxiety and harm avoidance (Hillert et al., 2013) as well as with absorption (Witthöft et al., 2008) which are all indices of reactivity in PARCS theory.

The present study does have some limitations. First, our study had a relatively small number of participants (*N* = 78). Second, although the ATQ provided suitable measures that have been used in related research before (Evans and Rothbart, 2008), no scales have yet been developed specifically to measure the constructs derived from PARCS theory. Third, our objective measure of sensitivity concerned auditory sensitivity only. Replication of our findings in a large sample using objective measures in other sensory modalities and using a questionnaire based on PARCS theory is important to show robustness and generalizability of these findings. In addition, future studies may also benefit from psychophysical measures of the response to strong stimuli, such as the threshold of pain and objective measures of reward and punishment reactivity such as ERP responses to reward and punishment stimuli during task performance (e.g., Boksem et al., 2006, 2008).

## Conclusion

Taken together, our study showed that self-reported as well as objectively assessed sensitivity to weak stimuli was associated with punishment and reward reactivity to strong stimuli. These relationships only became apparent when the reactivity measures were controlled for each other, indicating a mutual suppression effect. The fact that previous studies did not take this suppression effect into account may explain previous discrepant findings concerning the relation between sensory sensitivity and overreactivity. To conclude, our study indicates that sensitivity to weak stimuli overlaps, at least partly, with the tendency for overreactivity to strong stimuli, in a manner that is in line with the predictions of PARCS theory.

## Ethics Statement

This study was approved by the ethics committee of the Institute of Psychology of Leiden University. All participants gave written informed consent prior to participation.

## Author Contributions

All authors were involved in designing the study. AB performed the research. MT and AB analyzed the data. AB drafted the manuscript with input from GB, MT, and PS. All authors critically revised the manuscript and approved the final version for submission.

## Conflict of Interest Statement

The authors declare that the research was conducted in the absence of any commercial or financial relationships that could be construed as a potential conflict of interest.
