# Supplementary material for: Perceptual Sensitivity and Response to Strong Stimuli Are Related
Source: Front Psychol. 2017 Sep 22;8:1642. doi: 10.3389/fpsyg.2017.01642 (PMC5615480; doi:10.3389/fpsyg.2017.01642)
Supplement: Supplementary file 1 [file Table_1.pdf]

Supplementary Material

Perceptual Sensitivity and Response to Strong Stimuli are Related

Anna C. Bolders*, Mattie Tops, Guido P.H. Band, Pieter Jan M. Stallen

*** Correspondence:** Corresponding Author: anna.c.bolders@gmail.com

Table 1. *Pearson's correlation coefficients between the masked auditory threshold and the Adult Temperament Questionnaire (ATQ) scales*

	Masked Auditory Threshold	ATQ Negative Affectivity	ATQ Extraversion/ Surgency	ATQ Effortful Control	ATQ Orienting Sensitivity
Masked Auditory Threshold	1				
ATQ Negative Affectivity	.066	1			
ATQ Extraversion/Surgency	-.172	-.450**	1		
ATQ Effortful Control	.047	-.327**	.055	1	
ATQ Orienting Sensitivity	-.308**	.250*	.017	-.130	1

Note. * $p < 0.05$, ** $p < 0.01$

Table 2. *Pearson's correlation coefficients between the masked auditory threshold and the ATQ subscales*

	Masked Auditory Threshold	Fear	Frustration	Sadness	Discomfort	Activation Control	Attentional Control	Inhibitory Control	Sociability	High Intensity Pleasure	Positive Affect	Neutral Perceptual Sensitivity	Affective Perceptual Sensitivity	Associative Sensitivity
Masked Auditory Threshold	1													
Fear	.047	1												
Frustration	.124	.355**	1											
Sadness	.087	.282*	.154	1										
Discomfort	-.057	.385**	.305**	.192	1									
Activation Control	.076	-.116	-.225*	-.135	.152	1								
Attentional Control	-.041	-.216	-.272*	-.468**	-.194	.464**	1							
Inhibitory Control	.094	-.152	-.342**	-.155	-.067	.518**	.478**	1						
Sociability	-.110	-.193	-.185	-.156	-.178	.259*	.234*	-.056	1					
High Intensity Pleasure	-.152	-.294**	.037	-.312**	-.542**	-.323**	.016	-.172	.165	1_				
Positive Affect	-.072	-.176	-.169	-.060	-.094	.283*	.020	.075	.408**	-.052	1			
Neutral Perceptual Sensitivity	-.230*	.035	-.123	.133	.176	.063	-.004	.120	-.002	-.030	-.091	1		
Affective Perceptual Sensitivity	-.212	.119	.002	.341**	.292**	.027	-.244*	.055	.012	-.098	.124	.362**	1	
Associative Sensitivity	-.229*	.006	-.003	.166	.226*	-.142	-.238*	-.212	.034	-.040	.191	.196	.310**	1

Note. * $p < 0.05$, ** $p < 0.01$

Table 3. *Descriptive statistics for the ATQ scales (in bold) and subscales*

Scale	#Items	*M (SD)*	Skewness	Kurtosis	Cronbach's Alpha
**Negative affect scale (ATQ)**	**26**	**3.15 (0.57)**	**0.20**	**0.33**	**0.81**
Fear scale (ATQ)	7	2.92 (0.73)	0.20	-0.26	0.63
Frustration scale (ATQ)	6	3.30 (0.82)	0.41	0.41	0.67
Sadness scale (ATQ)	7	3.46 (0.80)	-0.08	0.16	0.74
Discomfort scale (ATQ)	6	2.92 (0.98)	0.56	-0.48	0.78
**Effortful control scale (ATQ)**	**19**	**3.56 (0.66)**	**-0.13**	**-0.39**	**0.80**
Activation Control scale (ATQ)	7	3.54 (0.80)	-0.37	-0.61	0.67
Attentional Control scale (ATQ)	5	3.45 (0.89)	-0.18	0.10	0.72
Inhibitory Controlscale (ATQ)	7	3.69 (0.73)	0.30	-0.32	0.49
**Extraversion/surgency scale (ATQ)**	**17**	**4.17 (0.56)**	**-0.25**	**0.27**	**0.68**
Socialbility scale (ATQ)	5	4.54 (0.84)	-0.47	-0.41	0.70
High Intensity Pleasure scale (ATQ)	7	3.46 (0.95)	0.01	-0.62	0.67
Positive Affect Scale (ATQ)	5	4.50 (0.73)	-0.22	-0.28	0.57
**Orienting sensitivity scale (ATQ)**	**15**	**3.73 (0.59)**	**0.22**	**-0.11**	**0.67**
Neutral Perceptual Sensitivity scale (ATQ)	5	3.92 (0.73)	0.19	-0.25	0.34
Affective Perceptual Sensitivity scale (ATQ)	5	3.68 (0.82)	0.11	-0.02	0.54
Associative Sensitivity scale (ATQ)	5	3.60 (0.87)	-0.04	-0.61	0.62
